# Capecitabine in the treatment of metastatic renal cell carcinoma

**DOI:** 10.1054/bjoc.2000.1340

**Published:** 2000-08-16

**Authors:** K Oevermann, J Buer, R Hoffmann, A Franzke, A Schrader, T Patzelt, H Kirchner, J Atzpodien

**Affiliations:** Department of Haematology and Oncology, Medizinische Hochshule Hannover, Carl-Neuberg-Strasse 1, Hannover, 30625, Germany; European Institute for Tumor Immunology and Prevention (EUTIP), Gotenstr. 152, Bonn, 53175, Germany; Robert Janker Cancer Center, Villenstr. 4, Bonn, 53129, Germany

**Keywords:** renal cell carcinoma, capecitabine, 13-cis-retinoic acid, alpha-interferon

## Abstract

To evaluate the therapeutic effects and systemic toxicities of a capecitabine-based home therapy regimen in patients with metastatic renal cell carcinoma, 30 patients were enrolled in a phase II clinical trial. Treatment consisted of oral capecitabine combined with subcutaneous recombinant human interferon-α 2a, recombinant human interleukin-2 and oral 13-cis-retinoic acid. There were two (7%) complete responses (CRs) and eight (27%) partial remissions (PRs), for an overall objective response rate of 34% (95% CI 17–53%). Except one, all responses are ongoing, with a median duration of 9+ and 8+ months for CRs and PRs, respectively. Additionally, 12 patients (40%) reached stable disease. Eight patients (27%) showed continued disease progression despite treatment. Therapy was well tolerated and was given in the outpatient setting. Capecitabine-related World Health Organization (WHO) grade 2 and 3 toxicities were observed in five and two patients respectively, and were limited to fatigue, nausea/vomiting, diarrhoea, stomatitis, dermatitis and hand-and-foot syndrome. The substitution of capecitabine for 5-FU in the pre-existing biochemotherapy regimen did not result in a reduced therapeutic efficacy and showed significant anti-tumour activity in patients with advanced renal cell carcinoma. © 2000 Cancer Research Campaign


British Journal of Cancer (2000) 83(5), 583–587
© 2000 Cancer Research Campaign
doi: 10.1054/ bjoc.2000.1340, available online at http://www.idealibrary.com on IDEAL®

### Capecitabine in the treatment of metastatic renal cell carcinoma

**K Oevermann[1], J Buer[1], R Hoffmann[1], A Franzke[1], A Schrader[1], T Patzelt[2], H Kirchner[1] and J Atzpodien[1,2,3]**

[1]Medizinische Hochshule Hannover, Department of Haematology and Oncology, Carl-Neuberg-Strasse 1, 30625 Hannover, Germany
[2]European Institute for Tumor Immunology and Prevention (EUTIP), Gotenstr. 152, 53175 Bonn, Germany
[3]Robert Janker Cancer Center, Villenstr. 4, 53129 Bonn, Germany

**Summary** To evaluate the therapeutic effects and systemic toxicities of a capecitabine-based home therapy regimen in patients with metastatic renal cell carcinoma, 30 patients were enrolled in a phase II clinical trial. Treatment consisted of oral capecitabine combined with subcutaneous recombinant human interferon-α 2a, recombinant human interleukin-2 and oral 13-cis-retinoic acid. There were two (7%) complete responses (CRs) and eight (27%) partial remissions (PRs), for an overall objective response rate of 34% (95% CI 17–53%). Except one, all responses are ongoing, with a median duration of 9+ and 8+ months for CRs and PRs, respectively. Additionally, 12 patients (40%) reached stable disease. Eight patients (27%) showed continued disease progression despite treatment. Therapy was well tolerated and was given in the outpatient setting. Capecitabine-related World Health Organization (WHO) grade 2 and 3 toxicities were observed in five and two patients respectively, and were limited to fatigue, nausea/vomiting, diarrhoea, stomatitis, dermatitis and hand-and-foot syndrome. The substitution of capecitabine for 5-FU in the pre-existing biochemotherapy regimen did not result in a reduced therapeutic efficacy and showed significant anti-tumour activity in patients with advanced renal cell carcinoma. © 2000 Cancer Research Campaign

**Keywords**: renal cell carcinoma; capecitabine; 13-cis-retinoic acid; alpha-interferon

Renal cell carcinoma is known to be mostly chemotherapy-resistant (Hrusshesky and Murphy, 1977; Duensing et al, 1994; Yagoda et al, 1995). While the prognosis of patients with metastatic renal cell carcinoma remains poor, objective and durable remissions can be reached in approximately one third of patients using palliative biochemotherapies, especially employing combined cytokines and 5-fluorouracil (Lopez et al, 1996; Ellerhorst et al, 1997).

Capecitabine is an orally administered fluoropyrimidine carbamate, activated by a three-step conversation process to the cytotoxic agent 5-FU. After oral administration, capecitabine is first metabolized in the liver to 5′-DFCR. Second, 5′-DFCR is converted to 5′-DFUR by cytidine deaminase, located in high concentrations in the liver and tumour tissues (Takebayashi et al, 1996; Miwa et al, 1998). Finally, conversation of 5′-DFUR to 5-FU occurs by thymidine phosphorylase, which is present at higher concentrations in tumour than in normal tissue, thus minimizing the exposure of healthy body tissues to 5-FU (Takebayashi et al, 1996; Ishikawa et al, 1998*a*; 1998*b*). The efficacy and favourable safety profiles of capecitabine have been demonstrated in patients with common solid tumours such as colorectal and breast cancer (Blum et al, 1999; Mackean et al, 1998; Schüller et al, 1997). So far, no results have been reported in advanced renal carcinoma.

Here we report a phase II clinical trial of p.o. capecitabine combined with s.c. interferon-α2a, s.c. interleukin-2 and p.o. 13-cis-retinoic acid. Systemic toxicity and therapeutic response were evaluated in 30 patients with progressive metastatic renal cell carcinoma. The results of this study are presented.

*Received 9 June 1999*
*Revised 10 May 2000*
*Accepted 17 May 2000*

*Correspondence to*: J Atzpodien

### PATIENTS AND METHODS

The study protocol was approved by the Clinical Institutional Ethical Review Board of the University of Hannover. Between June 1998 and December 1998 we entered 30 patients in a phase II clinical trial, (Table 1). All patients presented with histologically confirmed metastatic renal cell carcinoma in advanced state, aged 18–75 years, expected survival of more than 3 months, Karnofsky status of ≥ 70%, and signed informed consent.

Patients received the following capecitabine-based outpatient chemo-immunotherapy regimen. Interferon-α was administered subcutaneously at 5 MIU $m^{-2}$ on day 1 of weeks 5–8. Interleukin-2 was administered subcutaneously at 10 MIU $m^{-2}$ on days 3, 4 and 5 of weeks 1 and 4, and at 5 MIU $m^{-2}$ on days 1, 3 and 5 of weeks 2 and 3. Capecitabine was administered orally on days 1–5 of weeks 5–8 at 1000 mg $m^{-2}$ twice daily. In addition, oral 13-cis-retinoic acid was given at 34 mg $m^{-2}$ daily during weeks 1–8. Concomitant medication was given as needed to control adverse effects of immunotherapy. Patients were treated on an outpatient basis. Table 2 summarizes the therapy regimen used in this study.

8-week treatment cycles were repeated for up to three courses. Number of treatment cycles varied exclusively based on progressive disease of the patients. Re-evaluation of the patients' tumour status was performed between treatment cycles.

Response to therapy was evaluated according to World Health Organization (WHO) criteria: complete response = disappearance of all signs of disease for a minimum of 8 weeks; partial response = 50% or more reduction in sum of products of the greatest perpendicular diameters of measurable lesions, no increase in lesion size and no new lesions; stable disease = less than a partial response with no disease progression for at least 8 weeks; progressive disease = 25% and more increase in sum of the products in the

**Table 1** Patients characteristics

| Characteristics | Patients |
|---|---|
| Entered | 30 |
| Sex | |
| male | 24 |
| female | 6 |
| Age (years) | |
| median | 60 |
| range | 38–73 |
| Tumour nephrectomy | |
| yes | 29 |
| no | 1 |
| Metastases | |
| lung | 23 |
| bone | 14 |
| lymph nodes | 12 |
| liver | 9 |
| pleura | 4 |
| renal | 4 |
| adrenal gland | 3 |
| local relapse | 1 |
| other[a] | 5 |
| Prognosis[b] | |
| good | 5 |
| poor | 25 |
| No. of treatment cycles | 46 (range 1–3) |

[a]Tumour thrombus, soft tissue, pericardium, myelon ($n$ = 2); [b]Good prognosis was defined by the presence of lung metastases and the absence of bone metastases, ESR < 70 mm after 1 h, serum lactic dehydrogenase < 280 units $l^{-1}$, neutrophilic granulocytes < 6000 $\mu l^{-1}$ and haemoglobin > 100 gm $l^{-1}$ poor prognosis patients included all others longest perpendicular diameters of measurable lesions, or development of new lesions.

Systemic toxicity was evaluated at weekly intervals using a grading system adapted from the World Health Organization (WHO).

Treatment efficacy was assessed on intent-to-treat basis.

### RESULTS

### Median follow-up and survival

Median follow-up of all patients is 8 months; except for three patients, all patients are still alive.

### Treatment response

Out of 30 patients, two (7%) achieved a complete response, and eight (27%) had a partial remission (Table 3). The overall response rate in this study was 34% (95% CI 17–53%). Complete responses (CRs) occurred in lung ($n$ = 1), liver ($n$ = 1), pleura ($n$ = 1) and kidney ($n$ = 1), while partial remissions (PRs) were seen in lung ($n$ = 4), liver ($n$ = 3) (Figure 1), bone ($n$ = 1), pleura ($n$ = 1) and local relapse ($n$ = 1). The median response duration for CRs and PRs were 9+ and 8+ months, respectively, with a range from 4–10+ months (Table 4). Except for one patient who developed brain metastases, all patients are in sustained remission. In addition, 12 patients (40%) achieved stable disease upon chemo-immunotherapy (median duration of 8+ months). Eight patients (27%) had continued disease progression despite treatment.

### Toxicity

In all patients, capecitabine therapy was completed without modification of dosage or change of time-interval. 25 of 30 patients reported capecitabine-associated, mostly mild side-effects (Table 5). No grade 4 toxicity was observed and only two patients reported grade 3 malaise, one of them with concomitant grade 3 nausea/vomiting and stomatitis. Grade 2 stomatitis, dermatitis, hand-and-foot syndrome, anorexia, diarrhoea and malaise were observed in four, two, two, two, one and one patients, respectively. Mild grade 1 gastrointestinal side-effects were reported in 16, and mild anorexia in nine patients with moderate dermatitis, five showed early stages of hand-and-foot syndrome in the upper extremity and/or nail disorders. Few patients ($n$ = 9) developed grade 1 neurological symptoms including transient paraesthesiasis, headache and dizziness; eight patients reported mild myalgia. Grade 1 neutropenia was seen in one patient. No cardiac toxicity was noted; one patient with a history of severe aortic stenosis tolerated capecitabine without treatment-associated cardiac symptoms. In all patients treatment-related toxicities resolved after cessation of therapy. No toxic death occurred.

### DISCUSSION

The use of biologic therapies has an established role in the treatment of metastatic renal cell carcinoma (Quesada, 1988; Belldegrun et al, 1991; Atzpodien et al, 1995*a*; Hofmockel et al, 1997). Potentially better results can be obtained using combination therapies with cytokines and 5-fluorouracil (Lopez et al, 1996; Hofmockel et al, 1997). Retinoids are known to control many important biological processes, including differentation, morphogenesis, growth and tissue homeostasis (Warrel, 1994). Clinical and pre-clinical results provide evidence for an antiproliferative effect of 13-cis-retinoic acid in IFN-α-treated patients with renal cell carcinoma (Buer et al, 1995; Motzer et al, 1995). Furthermore, it seems to have a favourable effect in the treatment of renal

**Table 2** Home therapy regimen

| Therapeutic agent | Dose | Regimen |
|---|---|---|
| Recombinant interferon-α2 | 5 Million IU $m^{-2}$ s.c. | Once daily, day 1, weeks 1 and 4; days 1,3,5, week 2 and 3 |
| | 10 Million IU $m^{-2}$ s.c. | Once daily, days 1,3,5, weeks 5–8 |
| Recombinant interleukin-2 | 10 Million IU $m^{-2}$ s.c. | Twice daily, days 3,4,5, weeks 1 and 4 |
| | 5 Million IU $m^{-2}$ s.c. | Once daily, days 1,3,5, weeks 2 and 3 |
| Capecitabine | 1000 mg $m^{-2}$ p.o. | Twice daily, days 1–5, weeks 5–8 |
| 13-cis-retinoic acid | 35 mg $m^{-2}$ p.o. | Daily, days 1–7, weeks 1–8 |

Patients were entered between June 1998 and December 1998. Patients received cytokines and chemotherapy at home. Treatment cycles were repeated every 2 months for an average of 1.5 cycles (range 1–3) unless disease progression occurred

 © 2000 Cancer Research Campaign

**Table 3** Response to therapy

| Tumour site[a] | Response | | | | |
|---|---|---|---|---|---|
| | Complete remission | Partial remission | Stable disease | Progressive disease | Total |
| Lung | 1 | 6 | 10 | 6 | 23 |
| Bone | 1 | 3 | 7 | 3 | 14 |
| Lymph nodes | – | 2 | 4 | 6 | 12 |
| Liver | – | 4 | 2 | 3 | 9 |
| Pleura | 1 | 1 | 1 | 1 | 4 |
| Kidney | 1 | – | 1 | 2 | 4 |
| Adrenal gland | – | – | 2 | 1 | 3 |
| Local relapse | – | 1 | – | – | 1 |
| Other | – | 1[b] | 3[c] | 1[d] | 5 |
| Patients | 2 | 8 | 12 | 8 | 30 |
| Risk[e] *Good/Poor* | 1/1 | 2/6 | 2/10 | 0/8 | 5/25 |

[a]Patients may have had more than one tumour site; [b]Soft tissue; [c]tumor thrombus, pericardium, myelon; [d]myelon; [e]Good Prognosis was defined by the presence of lung metastases and the absence of bone metastases, ESR < 70 mm after 1 h, serum lactic dehydrogenase < 280 units $l^{-1}$ neutrophilic granulocytes < 6000 $\mu l^{-1}$ and haemoglobin > 100 gm $l^{-1}$, poor prognosis patients included all others

**Table 4** Remissions

| Age (years) | Sex | Interval between first diagnosis and appearance of metastases (months) | Sites of metastatic disease upon capecitabine, αINF, IL2, 13-cis-RA | Prognosis[a] upon start of therapy | Maximal response to capecitabine, αINF, IL2, 13-cis-RA | Remission duration (months) from start of capecitabine, αINF, IL2, 13-cis-RA | Status |
|---|---|---|---|---|---|---|---|
| 64 | m | 11 | Lung | Good | CR | 9+ | alive |
| 71 | f | 14 | Kidney, pleura, bone | Poor | CR | 9+ | alive |
| 41 | f | 0 | Lung, lymph nodes, liver, bone | Poor | PR | 4[b] | alive |
| 50 | m | 0 | Lung | Good | PR | 9+ | alive |
| 57 | m | 10 | Lung, liver, local relapse | Poor | PR | 8+ | alive |
| 57 | m | 25 | Bone, soft tissue | Poor | PR | 10+ | alive |
| 58 | m | 8 | Liver, lymph nodes | Poor | PR | 7+ | alive |
| 58 | m | 19 | Lung, liver, pleura | Good | PR | 8+ | alive |
| 64 | m | 23 | Lung | Poor | PR | 5+ | alive |
| 69 | m | 12 | Lung, bone | Poor | PR | 8+ | alive |

[a]Good prognosis was defined by the presence of lung metastases and the absence of bone metastases, ESR < 70 mm after 1 h, serum lactic dehydrogenase < 280 units $l^{-1}$ neutrophilic granulocytes < 6000 $\mu$ $l^{-1}$ and haemoglobin > 100 gm $l^{-1}$, poor prognosis patients included all others; [b]This patient developed brain metastases cancer, when added to immuno-chemotherapy regimens (Atzpodien et al, 1995*b*).

Capecitabine, as a novel fluoropyrimidine carbamate which is converted to 5-fluorouracil by three enzymes located in the liver and in tumours, shows promising response rates and remission durations while being very well tolerated in patients with metastatic renal cell carcinoma. Due to four-fold higher thymidine phosphorylase (5-DFUR to 5-FU) activity in tumour compared to adjacent healthy tissue (Frings, 1998), capecitabine allows for a more specific anti-tumour therapy than conventional i.v. fluorouracil.

In the present home-therapy trial, we treated 30 patients with progressive metastatic renal cell carcinoma with a combination of p.o. capecitabine, s.c. IFN-α, s.c. IL-2 and 13-cis-RA. The dose of capecitabine was adapted on an empirical basis from the recommend dose (Roche Laboratories Inc, 1998). The continued administration over an extended treatment interval of 4 weeks in combination with s.c. IFN-α, as opposed to an administration for 2 weeks followed by a 1-week rest period, required both a reduction in the daily dose of capecitabine and a > 50% reduction in cumulative 8-week capecitabine dosages. Capecitabine shows promising objective response rates in various solid tumours (Frings, 1998). We report first clinical results of capecitabine in the treatment of advanced renal cancer.

Tumour regressions occurred in 34% of patients evaluated, with 7% CRs, and median response duration of 8+ months. These results were comparable to other 5-FU-based therapy regimens in renal cell carcinoma (Sella et al, 1994; Joffe et al, 1996; Hofmockel et al, 1997; Tourani et al, 1998).

In the patients reported here, the rate of capecitabine-related toxicity was low, and side-effects were moderate overall. Predominant side-effects included gastrointestinal toxicities with diarrhoea, nausea/vomiting, dyspepsia and stomatitis, and cutaneous symptoms including dermatitis and early stages of hand-and-foot syndrome. A few patients experienced mild malaise and mild neurological/musculoskeletal side-effects.

Capecitabine-associated systemic toxicities were mostly limited to WHO grade 1, rarely grade 2. Only two patients experienced WHO grade 3 effects. It should be noted that in addition to capecitabine all patients were simultaneously treated with s.c. IFN-α and p.o. 13-cis-RA.

This confirmed the excellent tolerability reported in other malignancies at various dosages and treatment intervals of

*© 2000 Cancer Research Campaign* 

**Table 5** Systemic toxicity of capecitabine[a]

| Side-effects[b] (WHO criteria) | No. of patients | | | |
|---|---|---|---|---|
| | Grade 1 | Grade 2 | Grade 3 | Grade 4 |
| Gastrointestinal | 16 | 4 | 1 | – |
| Diarrhoea | 6 | 1 | – | – |
| Nausea | 15 | – | 1 | – |
| Vomiting | 7 | 1 | 1 | 2– |
| Stomatits | 7 | 4 | 1 | – |
| Abdominal pain | 7 | – | – | – |
| Constipation | 6 | – | – | – |
| Dyspepsia | 9 | – | – | – |
| Skin and subcutaneous | 17 | 2 | – | – |
| Hand-and-foot syndrome | 5 | 2 | – | – |
| Dermatitis | 17 | 2 | – | – |
| Nail disorder | 4 | – | – | – |
| General | 14 | 1 | 2 | – |
| Fatigue | 14 | 1 | 2 | – |
| Pyrexia | 7 | – | – | – |
| Neurological | 9 | – | – | – |
| Paraesthesia | 1 | – | – | – |
| Headache | 5 | – | – | – |
| Dizziness | 6 | – | – | – |
| Insomnia | – | – | – | – |
| Metabolism | 9 | 2 | – | – |
| Anorexia | 9 | 2 | – | – |
| Dehydration | 2 | – | – | – |
| Eye | 1 | – | – | – |
| Eye irritation | 1 | – | – | – |
| Muscoloskeletal | 8 | – | – | – |
| Myalgia | 8 | – | – | – |
| Cardiac | – | – | – | – |
| Oedema | – | – | – | – |
| Blood | 1 | – | – | – |
| Neutropenia | 1 | – | – | – |
| Thrombocytopenia | – | – | – | – |
| Anemia | – | – | – | – |
| Lymphopenia | – | – | – | – |
| Hepatobiliary | – | – | – | – |
| Hyperbilirubinaemia | – | – | – | – |

[a]Capecitabine associated toxicities were evaluated during weeks 5–8 of each treatment cycle according to WHO criteria and were observed in 25 of 30 patients. Grade 2 toxicity was seen in five patients and grade 3 toxicity occured in two patients; [b]Patients may have had several side-effects.

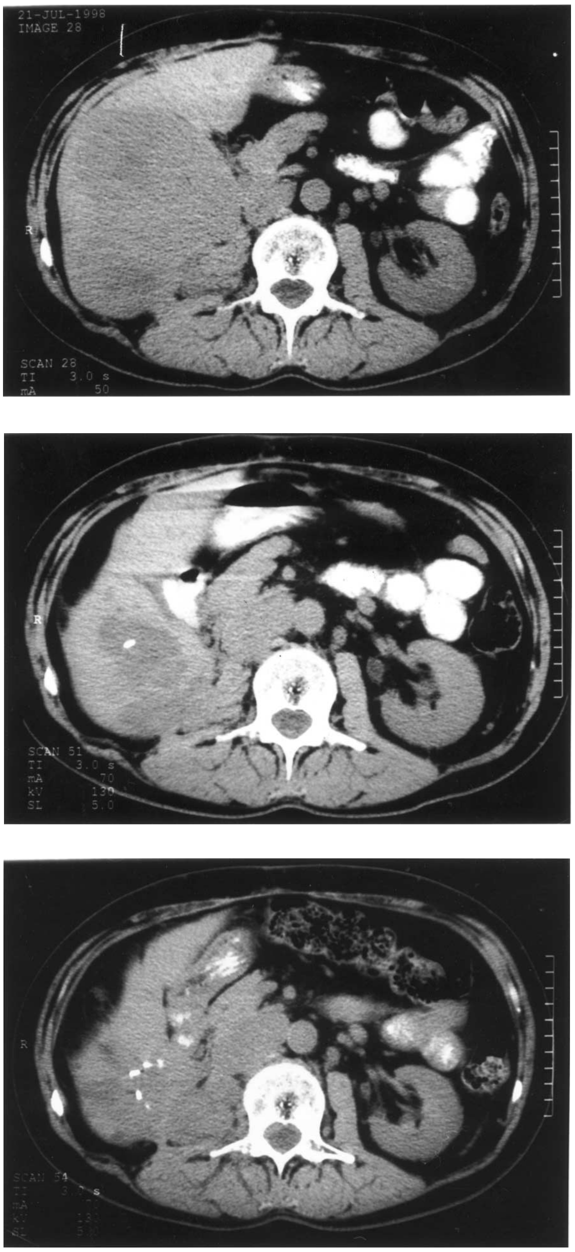

**Figure 1** Liver CT scans of a 58-year-old patient with a history of advanced renal cell carcinoma, post-surgical of tumour nephrectomy and partial liver resection who (**A**) subsequently developed extensive metastatic lesions in his liver. (**B**) Partial remission of metastatic liver disease after the first 8-week treatment cycle with p.o. capecitabine, s.c. INF-α; s.c. IL-2 and p.o. 13-cisRA. (**C**) Further tumour regression upon completion of the third consecutive treatment cycle.

capecitabine (Budman et al, 1998; Mackean et al, 1998). Per oral administration of capecitabine, in contrast to most chemotherapeutic agents that are applied intravenously, allows treatment in an outpatient or home therapy setting. This advantage will reduce expenses and could enhance quality of life in the palliative setting.

Unless randomized data become available, the contribution of capecitabine and its potential therapeutic feasibility cannot be definitively assessed. Therefore, we have initiated a prospectively controlled randomized clinical trial to investigate the role of capecitabine in patients with advanced renal cell carcinoma.

### ACKNOWLEDGEMENT

Jens Atzpodien and Jan Buer are supported by the Deutsche Krebshilfe.

### REFERENCES

Atzpodien J, Lopez HE, Kirchner H, Bodenstein H, Pfreundschuh M, Rebmann U, Metzner B, Illiger HJ, Jakse G and Niesel T (1995*a*) Multiinstitutional home-therapy trial of recombinant human interleukin-2 and interferon alfa-2 in progressive metastatic renal cell carcinoma. *J Clin Oncol* **13**: 497–501

Atzpodien J, Kirchner H, Duensing S, Lopez HE, Franzke A, Buer J, Probst M, Anton P and Poliwoda H (1995*b*) Biochemotherapy of advanced metastatic renal-cell carcinoma: results of the combination of interleukin-2, alpha-interferon, 5-fluorouracil, vinblastine, and 13-cis-retinoic acid. *World J Urol* **13**: 174–177

Belldegrun A, Abi-Aad AS, Figlin RA and deKernion JB (1991) Renal cell carcinoma: basic biology and current approaches to therapy. *Semin Oncol* **18**: 96–101

Blum JL, Jones SE, Buzdar AU, LoRusso PM, Kuter I, Vogel C, Osterwalder B, Burger HU, Brown CS and Griffin T (1999) Multicenter phase II study of

© 2000 Cancer Research Campaign capecitabine in paclitaxel-refractory metastatic breast cancer. *J Clin Oncol* **17**: 485–493

Budman DR, Meropol NJ, Reigner B, Creaven PJ, Lichtman SM, Berghorn E, Behr J, Gordon RJ, Osterwalder B and Griffin T (1998) Preliminary studies of a novel oral fluoropyrimidine carbamate: capecitabine. *J Clin Oncol* **16**: 1795–1802

Buer J, Probst M, Ganser A and Atzpodien J (1995) Response to 13-cis-retinoic acid plus interferon alpha2 in two patients with therapy refractory advanced renal cell carcinoma. *J Clin Oncol* **13**: 2679–2680

Duensing S, Dallmann I, Grosse J, Buer J, Lopez HE, Deckert M, Storkel S, Kirchner H, Poliwoda H and Atzpodien J (1994) Immunocytochemical detection of P-glycoprotein: initial expression correlates with survival in renal cell carcinoma patients. *Oncology* **51**: 309–313

Ellerhorst JA, Sella A, Amato RJ, Tu SM, Millikan RE, Finn LD, Banks M and Logothetis CJ (1997) Phase II trial of 5-fluorouracil, interferon-alpha and continuous infusion interleukin-2 for patients with metastatic renal cell carcinoma. *Cancer* **80**: 2128–2132

Frings S (1998) Capecitabine – a novel oral tumor activated fluoropyrimidine. *Onkologie* **21**: 451–458

Hofmockel G, Theiss M, Gruss A, Langer W and Frohmuller H (1997) Immunochemotherapy of metastatic renal cell carcinoma with interleukin 2, interferon-alpha and 5-fluorouracil. *Urologe A* **36**: 45–49

Hrusshesky WJ and Murphy GP (1977) Currents status of the therapy of advanced renal cell carcinoma. *J Surg Oncol* **9**: 277–288

Ishikawa T, Fukase Y, Yamamoto T, Sekiguchi F and Ishitsuka H (1998*a*) Antitumor activities of a novel fluoropyrimidine, N4-pentyloxycarbonyl-5′-deoxy-5-fluorocytidine (capecitabine). *Biol Pharm Bull* **21**: 713–717

Ishikawa T, Sekiguchi F, Fukase Y, Sawada N and Ishitsuka H (1998*b*) Positive correlation between the efficacy of capecitabine and doxifluridine and the ratio of thymidine phosphorylase to dihydropyrimidine dehydrogenase activities in tumors in human cancer xenografts. *Cancer Res* **58**: 685–690

Joffe JK, Banks RE, Forbes MA, Hallam S, Jenkins A, Patel PM, Hall GD, Velikova G, Adams J, Crossley A, Johnson PW, Whicher JT, and Selby PJ (1996) A phase II study of interferon-alpha, interleukin-2 and 5-fluorouracil in advanced renal carcinoma: clinical data and laboratory evidence of protease activation. *Br J Urol* **77**: 638–649

Lopez HE, Kirchner H and Atzpodien J (1996) Interleukin-2 based home therapy of metastatic renal cell carcinoma: risks and benefits in 215 consecutive single institution patients. *J Urol* **155**: 19–25

Mackean M, Planting A, Twelves C, Schellens J, Allman D, Osterwalder B, Reigner B, Griffin T, Kaye S and Verweij J (1998) Phase I and pharmacologic study of intermittent twice-daily oral therapy with capecitabine in patients with advanced and/or metastatic cancer. *J Clin Oncol* **16**: 2977–2985

Miwa M, Ura M, Nishida M, Sawada N, Ishikawa T, Mori K, Shimma N, Umeda I and Ishitsuka H (1998) Design of a novel oral fluoropyrimidine carbamate, capecitabine, which generates 5-fluorouracil selectively in tumours by enzymes concentrated in human liver and cancer tissue. *Eur J Cancer* **34**: 1274–1281

Motzer RJ, Schwartz L, Murray-Law T, Murphy BA, Hoffman AD, Albino AP, Vlamis V and Nanus DM (1995) Interferon alpha2 and cis-retinoic acid in renal cell carcinoma: Antitumor activity in a phase II trial and interactions in vitro. *J Clin Oncol* **13**: 1950–1957

Quesada JR (1988) Biologic response modifiers in the therapy of metastatic renal cell carcinoma. *Semin Oncol* **15**: 396–407

Roche Laboratories Inc. (1998) *Xeloda (capecitabine) prescribing information* Roche Laboratories: USA

Schüller J, Cassidy J and Reigner B (1997) Tumor selective activation of Capecitabine in colorectal cancer patients. *Onkologie* **20**: 189

Sella A, Kilbourn RG, Gray I, Finn L, Zukiwski AA, Ellerhorst J, Amato RJ and Logothetis CJ (1994) Phase I study of interleukin-2 combined with interferon-alpha and 5-fluorouracil in patients with metastatic renal cell cancer. *Cancer Biother* **9**: 103–111

Takebayashi Y, Akiyama S, Akiba S, Yamada K, Miyadera K, Sumizawa T, Yamada Y, Murata F and Aikou T (1996) Clinicopathologic and prognostic significance of an angiogenic factor, thymidine phosphorylase, in human colorectal carcinoma [see comments]. *J Natl Cancer Inst* **88**: 1110–1117

Tourani JM, Pfister C, Berdah JF, Benhammouda A, Salze P, Monnier A, Paule B, Guillet P, Chretien Y, Brewer Y, Di Palma M, Untereiner M, Malaurie E, Tadrist Z, Pavlovitch JM, Hauteville D, Mejean A, Azagury M, Mayeur D, Lucas V, Krakowski I, Larregain-Fournier D, Abourachid H, Andrieu JM and Chastang C Subcutaneous Administration Propeukin Program Cooperative Group. (1998) Outpatient treatment with subcutaneous interleukin-2 and interferon alfa administration in combination with fluorouracil in patients with metastatic renal cell carcinoma: results of a sequential nonrandomized phase II study. *J Clin Oncol* **16**: 2505–2513

Warrel RP (1994) Applications for retinoids in cancer therapy. *Semin Hematol* **31**(Suppl.): 1–13

Yagoda A, Abi-Rached B and Petrylak D (1995) Chemotherapy for advanced renal-cell carcinoma: 1983–1993. *Semin Oncol* **22**: 42–60

*© 2000 Cancer Research Campaign*
